# FGF-23 and Phosphate in Children with Chronic Kidney Disease: A Cross-Sectional Study in Kazakhstan

**DOI:** 10.3390/medicina57010015

**Published:** 2020-12-28

**Authors:** Altynay Balmukhanova, Kairat Kabulbayev, Harika Alpay, Assiya Kanatbayeva, Aigul Balmukhanova

**Affiliations:** 1 Department of Nephrology, Asfendiyarov Kazakh National Medical University, Almaty 050000, Kazakhstan; kairatkabulbayev@yahoo.com (K.K.); kanatbayeva@mail.ru (A.K.); 2 Division of Pediatric Nephrology, Marmara University, 34899 Pendik, Turkey; harika.alpay@gmail.com; 3 Department of Science and Innovations, Asfendiyarov Kazakh National Medical University, Almaty 050000, Kazakhstan; av.balmukhanova@gmail.com

**Keywords:** fibroblast growth factor 23, parathyroid hormone, hyperphosphatemia, phosphate metabolism, mineral and bone disorder

## Abstract

*Background and objectives:* Chronic kidney disease (CKD) in children is a complex medical and social issue around the world. One of the serious complications is mineral-bone disorder (CKD-MBD) which might determine the prognosis of patients and their quality of life. Fibroblast growth factor 23 (FGF-23) is a phosphaturic hormone which is involved in the pathogenesis of CKD-MBD. The purpose of the study was to determine what comes first in children with CKD: FGF-23 or phosphate. *Materials and Methods:* This cross-sectional study included 73 children aged 2–18 years with CKD stages 1–5. We measured FGF-23 and other bone markers in blood samples and studied their associations. *Results:* Early elevations of FGF-23 were identified in children with CKD stage 2 compared with stage 1 (1.6 (1.5–1.8) pmol/L versus 0.65 (0.22–1.08), *p* = 0.029). There were significant differences between the advanced stages of the disease. FGF-23 correlated with PTH (*r* = 0.807, *p* = 0.000) and phosphate (*r* = 0.473, *p* = 0.000). Our study revealed that the elevated level of FGF-23 went ahead hyperphosphatemia and elevated PTH. Thus, more than 50% of children with CKD stage 2 had the elevating level of serum FGF-23, and that index became increasing with the disease progression and it achieved 100% at the dialysis stage. The serum phosphate increased more slowly and only 70.6% of children with CKD stage 5 had the increased values. The PTH increase was more dynamic. *Conclusions:* FGF-23 is an essential biomarker, elevates long before other markers of bone metabolism (phosphate), and might represent a clinical course of disease.

## 1. Introduction

Nowadays, chronic kidney disease (CKD) has become a great medical and social problem in the health care system due to its very high morbidity and mortality both in Kazakhstan and around the world. The prevalence of CKD among populations both in Europe and Asia accounts over 10% and the number of patients is increasing gradually [1,2,3]. Pediatric epidemiology data are limited and vary in different countries. As reported in one study, the incidence of CKD in the pediatric population is approximately 11–12 per million of age-related population (pmarp) for stages 3–5, while the prevalence is about 55–60 pmarp [4]. According to Kanatbayeva, in Kazakhstan, the incidence and the prevalence of CKD among children and adolescents is higher than on average in the world [5].

It is well known that mineral and bone disorder (CKD-MBD) is one of the most serious complications, which might result in cardiovascular events, and might worsen the prognosis for patients [1,6]. As for children, there is an additional problem of growth and skeletal deformities because of calcium and phosphate metabolism disturbances [7,8].

Views on the pathogenesis of CKD-MBD have changed considerably since a novel phosphaturic hormone was discovered. Fibroblast growth factor 23 (FGF-23) is a 32 kDa peptide produced by osteocytes and osteoblasts. This 251-amino acids bone-derived protein includes a signal peptide, the N-terminal and the C-terminal interacting with α-Klotho [8,9,10]. FGF-23 induces stimulation of renal expression of phosphate due to direct suppression of sodium-phosphate co-transporters in the proximal tubules and inhibition of calcitriol production in kidneys [11]. As kidney function decreases, the level of FGF-23 concentration in serum gradually increases and reaches its maximum value in the end-stage kidney disease [12,13]. Moreover, positive correlations of FGF-23 with high mortality risk due to cardiovascular complications, as well as with independent left ventricular hypertrophy were reported in CKD adult populations [14,15,16]. Likewise, different correlations between FGF-23 and kidney function, calcium, phosphate, parathyroid hormone (PTH), and vitamin D have been shown in pediatric studies, which are limited in the literature [17,18,19,20]. Moreover, some of the studies have revealed controversial results. Studies that included children with CKD stages 1–2 are extremely scarce, making it difficult to identify early changes of FGF-23.

As no proven data on FGF-23 and phosphate metabolism in children in the Central Asia region are available, we decided to investigate this issue. The study aimed to investigate FGF-23 and phosphate metabolism in children living in a specific Asian region and to identify if FGF-23 moves at an earlier stage in children with CKD than phosphate and PTH.

## 2. Materials and Methods

### 2.1. Study Design and Participants

This single-center cross-sectional study was conducted on 73 children at the Nephrology and Dialysis Unit of University Hospital (Almaty, Kazakhstan) over the period from June to December 2019. Patients aged 2–18 years with the confirmed diagnosis of CKD stages 1–5 were eligible to be involved in the study except those who met the following exclusion criteria: children with tubulopathy, or active inflammatory, infectious, oncological, and bone diseases; those with a kidney transplant; as well as patients taking steroids, calcium, active vitamin D, or its supplements. We also compared FGF-23 concentration in patients with healthy controls, matched by age and gender (*n* = 14).

### 2.2. Ethics Approval

This study did not contradict the principles of the Declaration of Helsinki. The study protocol was approved by the Local Ethics Committee of the Asfendiyarov Kazakh National Medical University (No3 (80), 27 February 2019). Written informed consent was obtained from the parents of each participant.

### 2.3. Procedures and Laboratory Measurements

After physical examination, all relevant demographic, anamnestic and clinical information was recorded in the database. Laboratory tests were performed during the hospital stay of patients. The blood samples were obtained after 12 h of fasting.

CKD stage was defined according to KDIGO (Kidney Disease: Improving Global Outcomes) guidelines [21]. eGFR (estimated glomerular filtration rate) was calculated using the Schwartz Pediatric Bedside formula (2009). In accordance with the reference values of our laboratory, we determined hyperphosphatemia as the level phosphate in serum more than 1.8 mmol/L and hypocalcemia as serum calcium was less than 2.15 mmol/L. 25-OH vitamin D deficiency was defined as less than 20 ng/mL and insufficiency was defined as 20–30 ng/mL. Hyperparathyroidism was diagnosed according to normal ranges for each stage of CKD.

FGF23 (C-terminal) in serum concentration was assessed by a sandwich enzyme-linked immunosorbent assay (ELISA) kit (Biomedica Medizinprodukte GmbH, Vienna, Austria). FGF-23 concentration more than 1.5 pmol/L was considered as abnormal. Venous blood samples of investigation participants were collected in the standardized blood collection tubes and then centrifuged immediately for 20 min at 2000 rpm at 4 °C. After that, we aliquoted and stored the samples at −30 °C. All steps of laboratory assay were performed according to the manufacturer’s instructions. Then, a standard curve was constructed, and the sample concentration of FGF-23 was obtained from the standard curve.

### 2.4. Statistical Analyses

We used simple descriptive statistics. Normality distribution of data were analyzed according to the Kolmogorov–Smirnov test. Quantitative variables were expressed as mean ± standard deviation (SD); for variables within non-normal distribution we also calculated the median and interquartile range (IQR). We used Student’s *t*-test for unpaired samples in case of normal distribution. In other cases, we used non-parametric tests, such as the Mann–Whitney U test and Kruskal–Wallis test. A two-tail *p*-value of <0.05 was considered statistically significant. Spearman rank correlation method was applied to study the association between laboratory markers with CKD stages and other variables. All statistical analyses were performed using SPSS Statistics, version 22 (IBM Corp., Armonk, NY, USA).

## 3. Results

### 3.1. Patient Characteristics

The demographic and clinical characteristics of the study population are presented in Table 1. Investigation on primary causes of CKD revealed that there were 69.9% of children with congenital anomalies of the kidney and urinary tract (CAKUT), and 17.8%—with glomerular diseases. Four (5.5%) patients had nephrolithiasis and nephrocalcinosis, three patients (4.1%) had hemolytic uremic syndrome, and 2.7% of children had cystic kidney diseases as a primary diagnosis. Patients were divided into five groups according to CKD stages. We found that 28.8% of children had delay of growth and development, which is a real medical and social problem in children with CKD. Mineral-bone disorder was diagnosed as a CKD complication in 27 (37%) children. Renal anemia was found in over one third (34.2%) of all patients.

The data of laboratory evaluations of the patients in different stages of CKD are displayed in Table 2.

### 3.2. FGF-23 in Children with CKD

**Figure 1:**
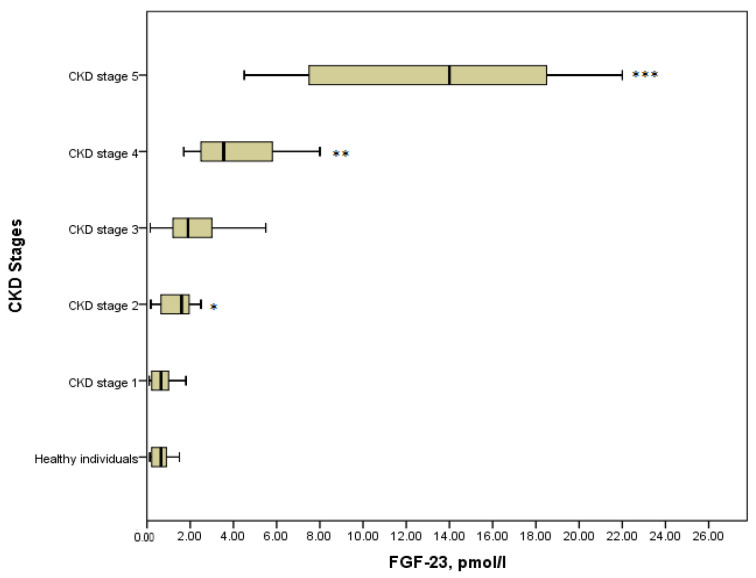
FGF-23 concentration in the various CKD stages (*p* = 0.000). * *p* = 0.029 CKD stage 2 vs. CKD stage 1; ** *p* = 0.021 CKD stage 4 vs. CKD stage 3; *** *p* = 0.001 CKD stage 5 vs. CKD stage 4.

It is obvious that the level of FGF-23 in serum elevated progressively across all stages of CKD (Figure 1). In healthy individuals median (IQR) serum FGF-23 was 0.65 (0.22–0.98) pmol/L, which did not differ statistically from CKD stage 1 with the median (IQR) value of 0.65 (0.22–1.08) pmol/L, (*p* = 0.8). It can be explained by the fact that stage 1 was asymptomatic and kidney function was absolutely intact. Further, a significant increase in the concentration of phosphaturic hormone could be noticed in stage 2 compared with the stage 1 patients (1.6 (1.5–1.8) pmol/L versus 0.65 (0.22–1.08), *p* = 0.029). Moreover, there were significant differences in the serum level of FGF-23 between the advanced stages of the disease. Thus, FGF-23 in children with CKD stage 4 showed a considerable increase concerning CKD stage 3 (3.55 (2.48–6.35) pmol/L versus 1.9 (1.15–3.5) pmol/L, *p* = 0.021). Similarly, the level of FGF-23 in serum in children on dialysis (14 (7.5–18.75) pmol/L) differed significantly from that in stage 4 (*p* = 0.001). In general, comparing all groups of patients with the Kruskal–Wallis test identified the statistical differences in the level of FGF-23, (*p* = 0.000) (Figure 1). 

**Figure 2:**
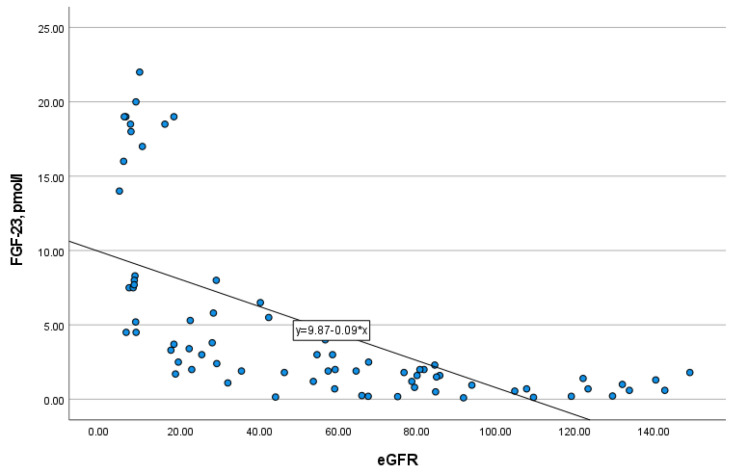
The correlation of FGF-23 with eGFR (*r* = −0.851, *p* = 0.000).

In addition, FGF-23 level strongly negatively correlated with eGFR (*r* = −0.851, *p* = 0.000) (Figure 2), and therefore, positively with CKD stages. The study of FGF-23 level in serum depending on the type of kidney replacement therapy (KRT) revealed that there were no differences between patients on hemodialysis and peritoneal dialysis (*p* = 0.772). Moreover, residual kidney function in patients of any type of KRT had no impact on the level of FGF-23 (*p* = 0.751).

There were no significant disparities in FGF-23 levels in patients with different etiology of CKD (*p* = 0.307). Thus, we decided to group all primary kidney diseases as glomerular and non-glomerular forms. As in the previous test, no statistically significant differences were found out (*p* = 0.075). Moreover, the serum concentration of FGF-23 between girls and boys did not differ significantly (*p* = 0.838). FGF-23 concentration was independent of age across all stages of CKD.

### 3.3. Phosphate, PTH and 25-OH Vitamin D in Children with CKD

**Figure 3:**
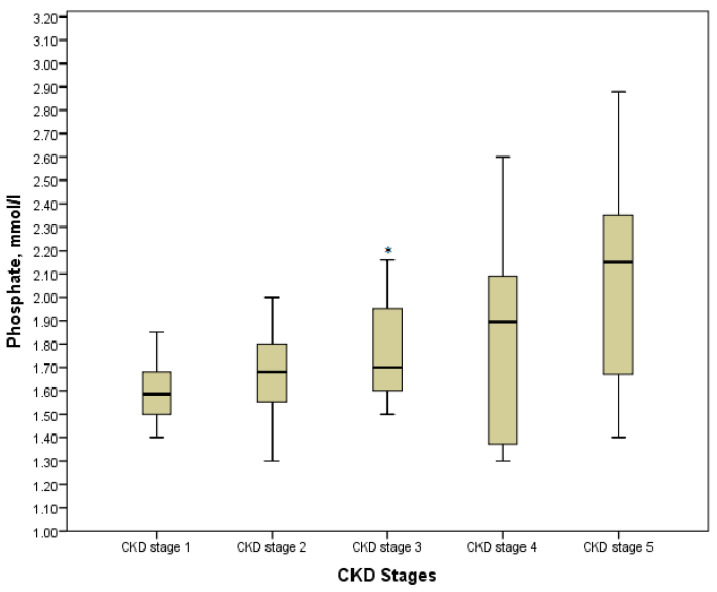
Phosphate level in the various CKD stages. * *p* = 0.013 CKD stage 3 vs. CKD stage 1.

According to the Kruskal-Wallis test results, phosphate, calcium, ionized calcium, PTH, and alkaline phosphatase in the explored groups with the various stages of the disease had statistically significant differences (*p* < 0.05). However, comparing the values in pairs of groups showed the controversial data. Thus, hyperphosphatemia was diagnosed in 32 (43.84%) children, especially in those who were pre-dialysis and dialysis with its maximum value of 2.88 mmol/L. On the other hand, a significant increase in phosphate was defined in stage 3 as compared with stage 1 (*p* = 0.013), whereas no statistically significant differences were found between values in stage 3 and stage 4, stage 4 and stage 5 (*p* = 0.716 and *p* = 0.085, respectively) (Figure 3). Moreover, we determined no differences between patients on hemodialysis or peritoneal dialysis (*p* > 0.05). The association between phosphate and CKD stages was significantly moderate (*r* = 0.425, *p* = 0.000).

Calcium and ionized calcium levels in serum fluctuated across all stages; however, CKD stage 5 drew attention by the prevalence of significant hypocalcemia. Thus, only 2 children of 17 patients KRT had a normal level of serum calcium.

**Figure 4:**
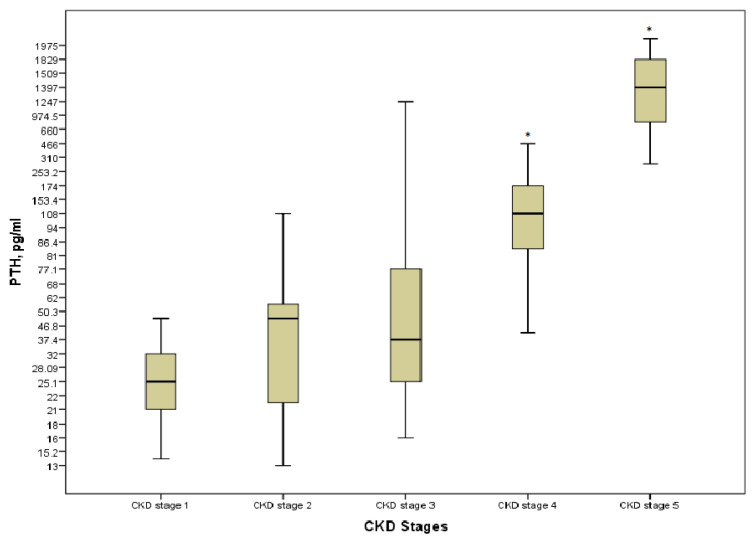
PTH level in the various CKD stages. * *p* = 0.000 CKD stages 4 and 5 vs. CKD stage 1.

Hyperparathyroidism was present in 30 (41.1%) patients; none of the patients on KRT had the target levels. Even though PTH was strongly positively correlated with stages of the disease (*r* = 0.828, *p* = 0.000), a significant increase was detected only at the pre-dialysis and the last stages of CKD (*p* = 0.000) (Figure 4). There were no differences in terms of the type of KRT (*p* > 0.05).

There were 23 (31.51%) children with a normal range of 25-OH vitamin D, 15 (20.55%) children suffered from vitamin D insufficiency, and 35 children (47.94%) had a deficiency of vitamin D. There was no significant association of 25-OH vitamin D with kidney function decline (*p* = 0.145).

### 3.4. Correlations of FGF-23 with Other Bone Markers

**Figure 5:**
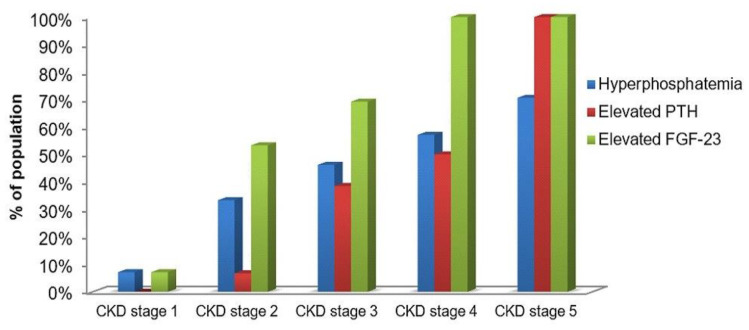
Proportion of study population with the elevated phosphate, PTH and FGF-23 in serum across all CKD stages (%).

As it is displayed in Figure 5, the elevated level of FGF-23 went ahead hyperphosphatemia and elevated PTH. The elevated FGF-23 was noticed in 7.1% of children with CKD stage 1. The same number of patients had increased indices of serum phosphate. It was remarkable that more than 50% of children with CKD stage 2 had the elevating level of serum FGF-23, and that index became increasing with the disease progression and it achieved 100% at the dialysis stage. Serum phosphate increased more slowly, and it was revealed only in 57.1% and 70.6% of children with CKD stage 4 and stage 5, respectively. About 7% of patients with stage 2 had elevated PTH. However, PTH increased more progressively and it was identified in 100% of the dialysis patients as well as FGF-23.

**Figure 6:**
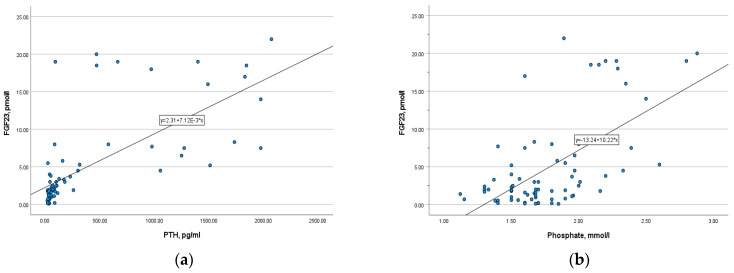
(**a**) The correlation of FGF-23 with PTH (*r* = 0.807, *p* = 0.000); (**b**) the correlation of FGF-23 with serum phosphate (*r* = 0.473, *p* = 0.000).

There was a strong positive correlation between phosphaturic hormone and PTH (*r*= 0.807, *p* = 0.000), as well as a moderate positive correlation with phosphate (*r* = 0.473, *p* = 0.000) (Figure 6). In opposite, FGF-23 level is inversely correlated with calcium, ionized calcium, and 25-OH vitamin D (*r* = −0.36, *p* = 0.002; *r* = −0.3, *p* = 0.009; *r* = −0.33, *p* = 0.004, respectively). The correlation was slightly weak.

## 4. Discussion

Our study is one of the few pediatric studies investigating the biomarkers of CKD-MBD. In accordance with the previous reports, we found out a progressive increase of FGF-23 with kidney function decline, i.e., from stage to stage [17,18,19,20]. However, even fewer pediatric studies have investigated the level of FGF-23 in early CKD, and moreover, the results were controversial. Thus, Van Husen et al. revealed in their study no significant differences in FGF-23 and other bone metabolism markers in patients with CKD stages 1 and 2 [19]. Likewise, in the report of Portale A. et al. the significant changes were defined only at stage 3 if compared with stage 2 [22]. In contrast, we studied CKD stage 1 and stage 2 separately and revealed statistically significant differences between the levels of FGF-23 in stage 2 and stage 1 (*p* < 0.05).

Furthermore, we calculated percentages of patients with phosphate, PTH and FGF-23 elevations in each stage. Even though phosphate gradually increases, these changes were not significant. The same findings were shown in other studies [17,19,22]. It makes us conclude that FGF-23 anticipates other bone markers that allows preventing many complications of CKD, including cardiovascular events, and life quality of children as well. On the other hand, 100% of CKD stage 5 patients had both elevated FGF-23 and PTH that makes it possible to use FGF-23 for diagnostics only at early stages.

Conflicting data about associations between FGF-23 and traditional laboratory markers of CKD-MBDhave been presented in different studies. We had a strong positive correlation of FGF-23 with PTH (*r* = 0.807, *p* = 0.000) and a moderate one with phosphate (*r* = 0.473, *p* = 0.000). Meanwhile, some authors revealed only a moderate correlation with PTH while others found a strong association with phosphate [18]. In our study we measured 25-OH vitamin D in patients and found no significant differences between all stages of CKD (*p* > 0.05). It occurs, probably, due to the high prevalence of 25-OH vitamin D insufficiency in our region regardless of renal function. In the study of Liu D. et al. FGF-23 was not correlated with 25-OH vitamin D, as it was revealed in our study [18]. By contrast, the interesting data were presented in one published study. The authors revealed no relationships between FGF-23 and serum phosphate and PTH whereas the decreased level of 25-OH vitamin D was associated with the elevated FGF-23 in serum [23].

It was thought that FGF-23 levels might depend on sex and age. However, no associations were found in our study (*p* = 0.838).

In the report of Portale A. et al., the authors postulated that glomerular etiology of CKD is associated with a higher level of FGF-23 than non-glomerular underlying causes of CKD [22]. Our data determined no significant differences in groups of glomerular and non-glomerular etiology (*p* = 0.075), as well as in groups with a particular primary diagnosis (*p* = 0.307).

Regarding a type of KRT, we found no relationships with any of the bone markers. Further, we wanted to evaluate whether residual kidney function might be an independent determinant of serum FGF-23 levels, as it was reported by Viaene L. et al. in an adult study population [24]. However, in our study we revealed neither FGF-23 nor traditional markers were associated with residual kidney function. These data should be investigated further in larger sample sizes and should require long term observation of patients under KRT.

The main limitation of our study is that it is a single-center cross-sectional study, and we were not able to observe patients for a long period of time. Moreover, the number of patients was limited. Therefore, we suggest the follow-up multi-central cohort studies involving more patients in order to implicate FGF-23 in clinical practice and to subsequently develop the new treatment targets in CKD-MBD.

## 5. Conclusions

In conclusion, we consider FGF-23 to be an essential marker, which increases along with kidney function declining and elevates long before other markers of CKD-MBD. It might be expected as one of the major laboratory variables reflecting a clinical course of CKD and used for early diagnostic of CKD-MBD.

Overall, to our knowledge, this is the first study among Central Asian countries, and specifically Kazakhstan, that was intended to investigate FGF-23 in children with CKD. The authors hope that the recent data extend knowledge in the field of pediatric nephrology and promote to further investigations.

## Figures and Tables

**Table 1:** Demographic and clinical data of the study population. CKD, chronic kidney disease; CAKUT, congenital anomalies of kidney and urinary tract; HUS, hemolytic uremic syndrome; HD, hemodialysis; PD, peritoneal dialysis; CKD-MBD, chronic kidney disease—mineral and bone disorder.

All Patients *(n* = 73)	
Age (year)	7.5 (4.0–11.0)
Female/male	35 (47.9)/38 (52.1)
CKD cause	
CAKUT	51 (69.9)
Glomerular diseases	13 (17.8)
Nephrolithiasis and nephrocalcinosis	4 (5.5)
HUS	3 (4.1)
Cystic kidney diseases	2 (2.7)
CKD stage	
Stage 1	14 (19.2)
Stage 2	15 (20.5)
Stage 3	13 (17.8)
Stage 4	14 (19.2)
Stage 5	17 (23.3)
HD	8 (11)
PD	9 (12.3)
Growth and development	
Normal	52 (71.2)
Delay	21(28.8)
CKD-MBD	27 (37)
Renal anemia	25 (34.2)

Data are presented as median (interquartile range (IQR)) or *n* (%).

**Table 2:** Laboratory variables of patients according to different stages of CKD. CKD, chronic kidney disease; eGFR, estimated glomerular filtration rate; PTH, parathyroid hormone; FGF-23, fibroblast growth factor-23.

Variable	Healthy Individuals	CKD Stage 1 eGFR >90 mL/min/1.73 m^2^ (*n* = 14)	CKD Stage 2 eGFR 60–89 mL/min/1.73 m^2^ (*n* = 15)	CKD Stage 3 eGFR 30–59 mL/min/1.73 m^2^ (*n* = 13)	CKD Stage 4 eGFR 15–29 mL/min/1.73 m^2^ (*n* = 14)	CKD Stage 5 eGFR <15 mL/min/1.73 m^2^ (*n* = 17)
Creatinine, mg/dL	N/A	0.4 ± 0.08	0.73 ± 0.19	1.11 (0.81–1.62)	2.32 ± 0.82	7.95 ± 2.43
eGFR, mL/min/1.73 m²	N/A	121.46 ± 17.94	77.17 ± 7.39	49.17 ± 9.61	22.5 ± 4.69	7.5 ± 1.55
Urea, mmol/L	N/A	3.68 ± 0.92	5.29 ± 1.44	6.5 (4.64–10.0)	14.48 ± 6.0	22.06 ± 4.6
Phosphate, mmol/L	1.52 ± 0.05	1.54 ± 0.21	1.66 ± 0.2	1.76 ± 0.21 ^a,f^	1.8 ± 0.41 ^a^	2.08 ± 0.45 ^a,c^
Calcium, mmol/L	N/A	2.23 (2.0–2.39)	2.1 (2.0–2.2)	2.16 (2.05–2.25)	2.15 (2.06–2.32)	1.8 (1.45–2.04) ^a,d^
Ionized calcium, mmol/L	N/A	1.2 (1.0–1.5)	1.2 (1.01–1.4)	1.25 (1.0–1.3)	1.26 (1.21–1.3)	1.1 (0.87–1.15) ^a,e^
Alkaline phosphatase, U/L	N/A	244.86 ± 74.7	300.76 ± 69.06 ^a^	250.56 ± 85.52	328.39 ± 119.95	495.6 ± 303.2 ^c^
PTH, pg/mL	25.5 (21.25–44.75)	25.5 (21.0–32.0)	46.8 (21.7–54.0)	37.4 (21.7–85.55)	110.34 (83.28–186.5) ^b,f^	1397 (814–1836) ^e,f^
25-OH vitamin D, ng/mL	N/A	29.4 ± 14.49	24.51 ± 10.64	21.16 ± 10.17	20.2 ± 14.37	20.08 ± 16.27
FGF-23, pmol/L	0.65 (0.22–0.98)	0.65 (0.22–1.08)	1.6 (1.5–1.8) ^a,f^	1.9 (1.15–3.5)	3.55 (2.48–6.35) ^c^	14.0 (7.5–18.75) ^d^

Data are presented as mean ± SD if normal distribution. Data are presented as median (IQR) if non-normal distribution. ^a^—*p* < 0.05, versus CKD stage 1. ^b^—*p* = 0.000, versus CKD stage 1. ^c^—*p* < 0.05, versus CKD stage 3. ^d^—*p* < 0.05, versus CKD stage 4. ^e^—*p* = 0.000, versus CKD stage 4. ^f^—*p* < 0.05, versus healthy individuals.

## Data Availability

The data presented in this study are available on request from the corresponding author. The data are not publicly available due to privacy and ethical issues.
